# Is There Evidence for Intelligence-by-Conscientiousness Interaction in the Prediction of Change in School Grades from Age 11 to 15 Years?

**DOI:** 10.3390/jintelligence11030045

**Published:** 2023-02-25

**Authors:** Anke Hufer-Thamm, Alexandra Starr, Ricarda Steinmayr

**Affiliations:** 1 Faculty of Psychology, Technical University Dortmund, 44227 Dortmund, Germany; 2 Department of Education, University of York, York YO10 5DD, UK

**Keywords:** school grades, educational success, intelligence-personality interaction, cognitive abilities, conscientiousness

## Abstract

Fluid intelligence and conscientiousness are the most important predictors of school grades. In addition to this main effect, researchers have suggested that the two traits might also interact with each other in the prediction of school success. A synergistic and a compensatory form of interaction have been suggested, but past evidence has been mixed so far. Most previous studies on this subject have been cross-sectional and many of them focused on older adolescents or adults in upper secondary school or university. We thus investigated the main and interaction effects of fluid intelligence and conscientiousness on school grades in math and German in a longitudinal sample of 1043 German students from age 11 to 15 years. Results from latent growth curve models with latent interaction terms showed a small compensatory interaction effect for baseline levels of math grades but not for their development. No interaction effect was found for German grades. These findings are discussed against the background that (synergistic) interaction effects between intelligence and conscientiousness might be more relevant in older students from higher secondary school or university context.

## 1. Introduction

Intelligence, particularly fluid intelligence, is the strongest single predictor of educational success, as it sets the frame for what a student is capable of achieving (Laidra et al. 2007; Mammadov 2022). Next to fluid intelligence, students’ personality explains a substantial amount of individual differences in educational achievement (e.g., Poropat 2009). Among the Big Five personality traits, conscientiousness has shown the strongest and most consistent associations with educational achievement such as school grades (Poropat 2009).

Apart from the impressive evidence for the unique contributions of fluid intelligence and conscientiousness in predicting success in school, these two factors may also interact meaningfully. Indeed, some studies have shown that intelligence and personality interact in the prediction of educational achievement (e.g., Bergold and Steinmayr 2018), whereas others could not find such an effect (Brandt and Lechner 2022; Zhang and Ziegler 2015). In addition, different lines of reasoning argue for two distinct interaction processes. The central research question here is whether students benefit from being intelligent *and* conscientious beyond the main effects of the traits or whether high conscientiousness can compensate for low cognitive abilities and vice versa.

To our knowledge, to date no study has investigated the potential longitudinal effects of intelligence-by-conscientiousness interactions on school grades, a highly relevant indicator of academic achievement, and could thus not model a potential interaction effect on the development of educational success. The majority of existing research also focuses on older students, using samples stemming from older adolescents or young adults from upper high school or university (but see, e.g., Brandt and Lechner 2022). The predictive value of intelligence, personality, and their interaction might be different in younger samples with a more diverse education background. To address these research gaps, in the present study we analyzed a longitudinal sample of German students between late childhood and mid-adolescence (age 11 to 15 years) in order to investigate the developmental effects of intelligence and personality on school grades in the domains of math and German.

### 1.1. Intelligence and Personality: Predictors of School Grades

General cognitive ability is a well-known predictor of academic achievement (Gottfredson 2002; Hofer and Clouston 2014; Neisser et al. 1996), especially when measured comprehensively including verbal (i.e., crystallized intelligence) and non-verbal (i.e., fluid intelligence) skills as shown in a meta-analysis by Roth et al. (2015). Fluid intelligence refers to the ability of problem-solving through abstract reasoning largely independent of prior learning, while crystalized intelligence captures accumulated skills and knowledge that are related to education and acculturation (Horn and Cattell 1967). Correlations with intelligence are higher for standardized achievement tests than for grades (Borghans et al. 2016; Deary et al. 2007; in comparison to Roth et al. 2015). The associations between standardized school performance and fluid intelligence tests range up to *r* = .74 in the population, with little variation across the domains of mathematics, science, and languages (cf. Rindermann 2006). With regard to school grades, research has shown that fluid intelligence is more strongly associated with math than with language (i.e., German; Brandt et al. 2020b). For mixed intelligence measures, the association is even stronger and remains statistically significant when tested longitudinally in adolescence (age 11–16). Here, intelligence explains about 60% of the variance in a standardized final exam across subjects (Deary et al. 2007). 

Apart from intelligence, personality characteristics are predictive of educational outcomes. In terms of the Big Five domains (McCrae and Costa 1987), meta-analyses by Poropat (2009) and Mammadov (2022) have demonstrated that conscientiousness is the most powerful predictor of school grades after controlling for cognitive abilities. Whereas conscientiousness was an important predictor at all educational stages, agreeableness and openness were more predictive during elementary school (see also, Andersen et al. 2020). Furthermore, recent studies have demonstrated that openness is more important for academic achievement operationalized via standardized scholastic achievement tests whereas conscientiousness is more predictive of grades (Hübner et al. 2022; Meyer et al. 2022; Tetzner et al. 2020). Indeed, further studies have confirmed medium correlations between self-reported grades (Cucina et al. 2016; Dumfart and Neubauer 2016) or teachers’ evaluations of school performance (Demetriou et al. 2019) and conscientiousness. Moreover, conscientiousness has shown stronger relations with math grades than with grades in the language domain (Brandt et al. 2020b). Findings highlight that high levels of conscientiousness—comprising diligence, good impulse control and self-regulation, the ability to persevere on challenging tasks, and organizing work efficiently—seem to be beneficial for academic success above and beyond one’s cognitive ability, especially when performance is assessed through grades. From a longitudinal perspective, there is first evidence that changes in school grades and conscientiousness correlate with each other (Israel et al. 2022). 

### 1.2. Fluid Intelligence, Conscientiousness, and School Grades in Adolescence

Adolescence is a crucial phase with regard to the development of personality and cognitive abilities (e.g., Soto and Tackett 2015). It is known to also be associated with development in related domains, thus changes might be relevant in the context of individuals’ achievement trajectories (e.g., Israel et al. 2022; Bardach et al. 2023). 

While personality is generally rather stable over time (e.g., Wrzus and Roberts 2017), changes in all personality traits occur across an individual’s lifespan with personality change being most pronounced in adolescence (i.e., rank-order stability; Roberts and DelVecchio 2000). In addition, personality development is also observable in terms of mean-level changes and in accordance with the maturity principle (Roberts et al. 2006), hence individuals’ conscientiousness is typically lower in early life compared to later stages. Previous findings show that dips in personality maturity are common in early adolescence followed by rapid increases into early adulthood (Denissen et al. 2013), making adolescence a particularly relevant period to study regarding personality development.

Levels of intelligence during this phase have shown to be rather stable overall from adolescence onwards (Deary et al. 2013; Yu et al. 2018), though individual differences in cognitive developmental trajectories are apparent in earlier periods associated with a variety of physiological as well as environmental factors (e.g., Shaw et al. 2006; von Stumm and Plomin 2015). While intelligence is commonly conceptualized as an antecedent of school achievement, a meta-analysis by Peng et al. (2019) highlights the complexity of the developmental relationship in terms of reciprocal associations between both constructs over time in adolescence.

### 1.3. Intelligence-by-Conscientiousness Interactions in the Prediction of School Grades

Research has long viewed and investigated intelligence and personality, especially conscientiousness, as independent predictors of academic success. However, some authors have proposed that the distinction between ‘cognitive’ and ‘non-cognitive’ characteristics is no longer valid and that the integration of the two entities into common frameworks is due (e.g., DeYoung 2020). Beyond well-established main effects, intelligence and conscientiousness may also interact with each other in a meaningful way to predict success in school. Two different forms of intelligence by personality interactions have been proposed: the *synergistic* and the *compensatory* interaction. In line with the particular relevance of conscientiousness to the prediction of school grades, we will focus on the interplay between fluid intelligence and conscientiousness in the following study (but see, e.g., Nießen et al. (2020), who found interaction effects of agreeableness and cognitive ability on success in educational transitions). With regard to conscientiousness, the synergistic interaction implies that high cognitive abilities and high conscientiousness amplify each other, while the compensatory interaction means that a student can make up for lower intelligence by being more diligent, and vice versa. Both of these forms have theoretical and empirical foundations, which we will summarize in the following.

#### 1.3.1. Synergistic Interaction: Intelligence and Conscientiousness Reinforcing Each Other?

The idea of trait-by-trait interactions for the prediction of performance is already present in older motivation theories from the field of organizational psychology (e.g., Maier 1965). These studies assumed an ordinal interaction between intelligence and motivation in the sense that high motivation should lead to a stronger relationship between intelligence and motivation, and vice versa (Sackett et al. 1998). Later work has transferred this approach to conscientiousness and the field of academic achievement (e.g., Bergold and Steinmayr 2018).

As another line of research, investment theories (e.g., Ackerman 1996; Cattell 1987; Ziegler et al. 2009) also assume an interplay of personality and fluid intelligence on the acquisition of knowledge and competencies (i.e., crystallized intelligence). Within this perspective, investment traits are “personality characteristics that determine where, when and how people apply their mental capacity” (von Stumm et al. 2011, p. 576). The predominant traits of interest in this field have been openness and related traits, such as the need for cognition (Cacioppo and Petty 1982) and epistemic curiosity (Litman 2008), while conscientiousness is usually not included in these studies. Very recently, Brandt and Lechner (2022) have made the case that conscientiousness encompasses characteristics of an investment trait that influence how much effort and fluid intelligence are invested in the acquisition of knowledge, such as persistence and self-discipline. Following this argument, conscientiousness should not only have a main effect on educational success but it should also amplify the effect of intelligence. Comparably, the invest-and-accrue model of conscientiousness (Hill and Jackson 2016) states that conscientiousness as a basic disposition drives investment-oriented behavior. With regard to educational achievement, individuals might choose learning activities over short-term rewards in order to accrue long-term benefits. 

In the concrete context of school success, being assiduous enables students to get the most out of their cognitive potential in the sense that the two characteristics reinforce each other and that only individuals with high levels of both characteristics will achieve the highest grades and make the strongest improvements. Conversely, when intelligence is low, a conscientious working style should matter less in achieving good grades (Bergold and Steinmayr 2018). Technically, if a synergistic interaction is present, the relationship between intelligence and educational success is stronger when levels of conscientiousness are high and is smaller at the lower end of the conscientiousness continuum.

Bergold and Steinmayr (2018) investigated the relationships between all Big Five personality traits, general mental ability, and GPA in two samples of students attending grade 11 in German upper secondary schools. They found that on domain level, both conscientiousness and openness predicted achievement beyond intelligence, but only conscientiousness displayed a statistically significant interaction with intelligence. The interactions between intelligence and conscientiousness were synergistic in the sense that the predictive value of cognitive ability was even stronger in students scoring highly on conscientiousness. On the other end of the ability continuum, intelligence was less predictive of grades for students who displayed lower levels of conscientiousness. In a very large pooled sample of German students from upper secondary schools, Meyer et al. (2022) recently found evidence for synergistic interactions predicting both standardized tests and grades, albeit with small effect sizes. Sorjonen et al. (2021) also noted that most of the personality-by-intelligence interactions in the prediction of academic achievement (i.e., GPA in an adult sample) were synergistic in nature, especially with regard to conscientiousness and related traits. Evidence for synergistic interaction effects of intelligence and conscientiousness also came from studies using samples of undergraduate students (Di Domenico and Fournier 2015; Ziegler et al. 2009). On the other hand, Brandt and Lechner (2022), who investigated knowledge gains in reading and math in standardized achievement tests in two samples of students (from grade 4 to grade 7 and from grade 7 to grade 9) found a synergistic intelligence-by-conscientiousness interaction, but only when control variables were not included.

#### 1.3.2. Compensatory Interaction: Intelligence and Conscientiousness Making up for Each Other?

Intelligence and conscientiousness are sometimes negatively correlated (e.g., Moutafi et al. 2004), although they both relate positively to achievement indicators. The compensatory interaction hypothesis helps explain this irregularity. According to this interaction form, less bright students can achieve good grades when they display a high level of conscientiousness, resulting in an advantageous working style. Likewise, higher intelligence might help students with lower conscientiousness make up for their carelessness. 

Chamorro-Premuzic and Furnham (2004) argued that students equipped with lower cognitive abilities might develop higher levels of conscientiousness in order to compensate for this deficit and to achieve comparable grades as their smarter classmates. Others (e.g., Murray et al. 2014), however, have put forth that the negative relation of cognitive abilities and conscientiousness could be due to a selection effect that vanishes in samples unselected with regard to academic achievement. Nevertheless, this explanation also assumes compensatory interaction, even though the underlying mechanism is different (Harris-Watson et al. 2022). 

Considerably fewer studies have yielded evidence for a compensatory intelligence-by-conscientiousness interaction in the prediction of academic success than for the synergistic interaction form. Evidence is provided by research on interaction effects in the prediction of (job) task performance (e.g., Harris-Watson et al. 2022; but see also van Iddekinge et al. 2018). Nevertheless, there are also studies that found neither synergistic nor compensatory interaction effects of intelligence and conscientiousness for the prediction of standardized tests or school grades (Heaven and Ciarrochi 2012; Zhang and Ziegler 2015).

## 2. The Current Study

In sum, despite the unequivocal importance of intelligence, empirical evidence of the predictive value of intelligence-by-conscientiousness interactions is inconsistent. Our study addressed a couple of aspects. First, most analyses are based on students in late adolescence or early adulthood who received their grades in the academic track of high school or in university or college (e.g., Meyer et al. 2022; Di Domenico and Fournier 2015). It is therefore necessary to investigate samples that include not only academic track students. As schooling is only mandatory up to a certain grade, and afterwards selection occurs resulting from changing mandatory to voluntary schooling, it is expedient to investigate younger samples of an age when schooling is still mandatory. In addition, young and middle adolescence is a crucial time with respect to personality and cognitive development. Second, most studies relied on cross-sectional data (e.g., Bergold and Steinmayr 2018; Meyer et al. 2022, but see Brandt and Lechner 2022) that impeded causal conclusions and the examination of the development of academic success over time. Both intelligence and conscientiousness (and their interaction) might not only predict academic success at one given time point, but also *changes* therein. Third, conscientiousness is considered a better predictor of grades than of performance in academic achievement tests (Hübner et al. 2022). Thus, it might well be that Brandt and Lechner (2022) were not able to find evidence for a statistically significant interaction of intelligence and conscientiousness (when including control variables) as they used standardized achievement tests and not grades as academic achievement indicators. To our knowledge, there is no study investigating the potential interaction effects of intelligence and conscientiousness in the prediction of the development of school grades in young adolescence when school is still mandatory.

The aim of the present study is to shed further light on the interplay of intelligence and conscientiousness and their prediction of school grades and their development. Unlike Brandt and Lechner (2022), who analyzed competence gains in reading and math as outcomes, we focused on school grades because grades are of higher relevance for the students’ lives than competences are as they are imperative for vocational success and subsequently predict social inequality in later life (Kingston et al. 2003). Additionally, based on the findings and argumentation of, for instance, Hübner et al. (2022) and Meyer et al. (2022), we assume that conscientiousness is more strongly associated with grades than with standardized test scores because grades are more dependent on teachers’ evaluations and a conscientious working style helps to fulfill the classroom requirement. It should thus be more likely to find statistically significant interaction effects for the prediction of school grades.

In the current study, we investigated the impact of fluid intelligence and conscientiousness on the development of school grades (math and German as first language) in a representative sample of German students over three measurement times from late childhood to middle adolescence (11 to 15 years). For the prediction of school grades by conscientiousness and intelligence, very solid evidence allows the formulation of specific hypotheses, whereas evidence regarding the interaction between intelligence and conscientiousness is not as consistent. Thus, the following not preregistered hypotheses (H) and research questions (RQ) guided our analyses:

**H1a.**  *Fluid intelligence and conscientiousness statistically significantly predict baseline levels of school grades. We expect the effects to be larger for math than for German.*

**H1b.**  *Fluid intelligence and conscientiousness significantly predict changes in school grades. We expect the effects to be larger for math than for German.*

RQ1a: Do intelligence and conscientiousness interact in the prediction of the *baseline level* of school grades?

RQ1b: Do intelligence and conscientiousness interact in the prediction of *changes* in school grades?

RQ2: If there are statistically significant interaction effects on level or development of school grades, are they synergistic or compensatory in nature?

## 3. Materials and Methods

### 3.1. Procedure and Participants 

We used data from TwinLife, a longitudinal, cross-sequential twin family study representative of the German population (Hahn et al. 2016; Lang and Kottwitz 2020). TwinLife set out to investigate the genetic and environmental processes in the development of social inequality and life chances. In total, the project comprises more than 4000 twin families in four twin birth cohorts (born in 2009/2010, in 2003/2004, in 1997/1998, and in 1991/1992, respectively) who participate in annual surveys conducted by an external institute alternatingly via face-to-face and telephone interviews starting in 2014. The sample of the current study contained data of the second youngest age cohort of the project (birth years 2003 and 2004) with a total sample size of 1043 same-sex twin pairs. We randomly selected one twin per family to derive an independent sample. 

At the first measurement occasion (t_1_, 2014–2016), the students were on average 11.00 years old (*SD* = 0.32) and 52.0% of the participants were female. The two other measurement times followed after two years each (t_2_: 2016–2018; t_3_: 2018–2020). In addition to the usual panel attrition, a change in the survey institute between t_1_ and t_2_ resulted in a substantial dropout from the first measurement occasion to the second (Brix et al. 2017; Lesaar et al. 2020). Table 1 depicts sample descriptions for all three measurement occasions. With regard to school type, at t_1_, 46.7% of the students of whom photos of the report cards were available were in elementary school, 20.6% in lower, intermediate level or comprehensive secondary school, and 27.5% in upper secondary school. At t_2_, 41.1% were in lower, intermediate level or comprehensive secondary school and 51.7% in upper secondary school. At t_3_, 2.4% were in schools for children with special needs, 39.7% in lower, intermediate level or comprehensive secondary school, and 54.8% in upper secondary school (the remainder was in other school types; more details can be found in the Supplementary Materials).

### 3.2. Measures

#### 3.2.1. School Grades

Information on school grades and the type of school attended was derived from students’ most recent report cards via photographs taken by the interviewer during the face-to-face interviews at all three measurement occasions upon parental consent. Report cards were available for 58.96% at t_1_. Students for whom we did not have indicators of grades did not differ statistically significantly from those with photos of their report cards with regard to conscientiousness (*F* = 0.464, *p* = .496; *t* = 0.415, *p* = .678) and intelligence (*F* = 0.001, *p* = .982; *t* = −1.157, *p* = .117). This information was then transferred into a coding scheme to account for differences between states and schools (for a detailed description, see Instinske et al. 2022). Subsequently, the variable was regressed on the type of school attended at each measurement point to account for potential bias in grading (elementary school vs. upper secondary school vs. other types of secondary school) resulting in a corrected variable. To ease interpretability, we inverted these variables for the main analyses so that higher values reflected better performance.

#### 3.2.2. Conscientiousness

Personality in terms of the Big Five dimensions (McCrae and Costa 1987) was assessed via self-reports at t_1_ using a short version of the Big Five Inventory (BFI-S; Gerlitz and Schupp 2005) comprising 16 items, three of which measure the dimension of conscientiousness (“I see myself as someone who… does a thorough job; tends to be lazy. (inversely coded); does everything efficiently.”). Participants rated each item on a 7-point rating scale (1 = *Does not apply to me at all*–7 = *Applies to me perfectly*). The items represented the indicators for the latent factor of conscientiousness. The internal consistency of the conscientiousness scale was ω = .538 in our sample.

#### 3.2.3. Fluid Intelligence

We measured fluid intelligence at t_1_ using four subtests (figural reasoning, figural classification, matrices, and reasoning) from the Culture Fair Test (CFT 20-R; Weiß 2006). Participants completed the test via a computer-based version (for details, see Gottschling 2017). The total sum scores of all four subtests were used for the latent factor. The measure had an adequate internal consistency (ω = .718) in the present sample.

#### 3.2.4. Socioeconomic Status (SES)

For parental SES, we generated a composite measure based on information on educational level, occupational status, and income. Parental educational level reflected the highest level of education attained as indicated by the Comparative Analysis of Social Mobility in Industrial Nations (CASMIN; Lüttinger and König 1988). Parental occupation was scaled by the International Socioeconomic Index of Occupational Status (ISEI; Ganzeboom et al. 1992) and the Erikson-Goldthorpe-Portocarero Class Schedule (EGP; Erikson et al. 1979). For CASMIN, ISEI, and EGP, the maximum of maternal and paternal values was used as the family index. Income at the family level was measured as the net household income (modified according to the OECD equivalence scale; see Hagenaars et al. 1998). The variable was winsorized (top and bottom 1%) to account for extreme outliers. All SES indicators were standardized and subsequently aggregated into a composite score at the family level, which we used for the following analyses.

### 3.3. Analyses

The main analyses were conducted using Mplus 8.5 (Muthén and Muthén 1998–2017). The respective codes are available on OSF (https://osf.io/vytg7/?view_only=159c84bb4a124c8d9b2871d5f8ef180b). Analyses were not preregistered. We applied latent growth curve (LGC; e.g., Bainter and Howard 2016) models using the maximum likelihood estimator with robust standard errors to model the development of the school grades over time, for which either grades in math or German were measured as manifest variables. Intelligence and personality traits were modeled as latent variables and were centered by restricting their mean to zero. We used the full information maximum likelihood algorithm to account for missing data. As robustness checks, we also conducted latent change models (see Supplementary Materials).

**Figure 1:**
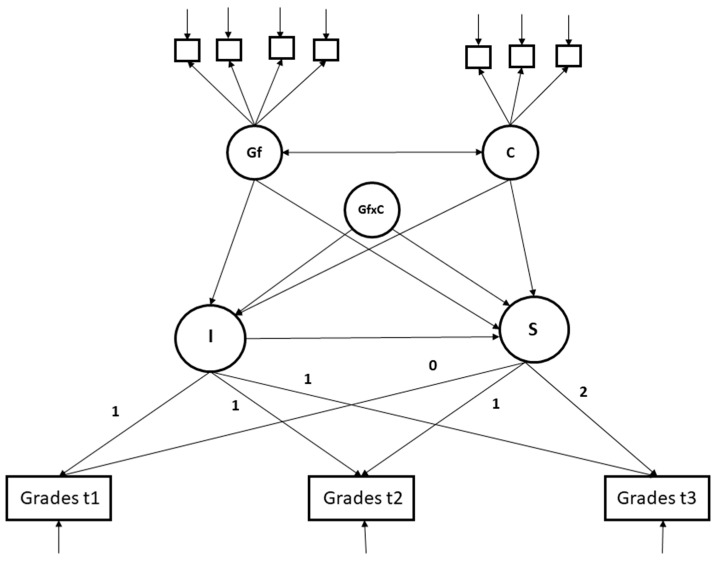
Schematic depiction of the latent growth curve model with the latent interaction term. *Note:* Gf = fluid intelligence; C = conscientiousness; GfxC = latent interaction between fluid intelligence and conscientiousness; I = intercept factor; S = slope factor. Control variables are omitted for simplicity.

In a first step, we modeled intelligence and conscientiousness as predictors of the intercept and the slope factor to estimate their relation with both the baseline level of school grades and changes in school grades. We included gender (1 = male/2 = female) and SES as covariates. Intelligence and conscientiousness were modeled as latent factors with four (CFT subtests) and three (BFI-S items) indicators. Because students who took part in the second measurement occasion had better grades and better intelligence test results at t_1_, we included participation at t_2_ as an auxiliary variable (0 = no/1 = yes) into the final baseline model. To answer our research questions, we used moderated structural equation modeling (Klein and Moosbrugger 2000) and added the latent interaction between intelligence and conscientiousness to the model. Figure 1 is a schematic depiction of the latent interaction model. 

## 4. Results

### 4.1. Descriptive Statistics and Correlations

In Table 1, means and standard deviations of manifest fluid intelligence and conscientiousness are depicted along with the internal consistency of the conscientiousness scale (McDonald’s ω). Table 2 shows the frequencies of math and German grades. Table 3 shows the correlations among all variables of interest. Girls were more conscientious than boys and had better German grades, but not math grades. In line with our expectations, both types of grades were positively related to fluid intelligence and conscientiousness at all measurement points, whereas intelligence and conscientiousness were uncorrelated.

### 4.2. Latent Growth Analyses: Predicting Level and Development of School Grades

In the univariate LGC models without predictors and covariates (see Table 4), the intercepts showed statistically significant interindividual variability. The slope factors implied a statistically significant change in grades, but no interindividual variance in the trajectories.

In the models with intelligence and conscientiousness as predictors of the intercepts and the slopes of school grades, but without the latent interaction term, we allowed SES and intelligence to correlate according to modification indices, which is theoretically plausible and notably increased the model fit (∆χ^2^ = 154.514; ∆RMSEA = .036; ∆CFI = .105 for math; ∆χ^2^ = 151.981; ∆RMSEA = .035; ∆CFI = .104 for German). We thus applied the same modification to the latent interaction model. The evaluation of the overall model fit followed established guidelines (Hu and Bentler 1999). Parameter estimates and model fit indices for these two models are depicted in Table 5. The inclusion of the auxiliary variable participation at t_2_ did not noticeably change the results.

For the latent interaction model, no indices of overall model fit (i.e., RMSEA, CFI or SRMR) have been developed (Maslowsky et al. 2015; Klein and Moosbrugger 2000), so we interpreted the Akaike information criterion (AIC) and the sample-size adjusted Bayesian information criterion (nBIC). In the latent interaction model, both fluid intelligence and conscientiousness statistically significantly predicted baseline levels in school grades (i.e., the intercept; H1a) but not their development (i.e., the slope; H1b). As hypothesized, the effect was larger for math grades (Table 4, part a) than for German grades (Table 4, part b). We found no statistically significant interaction effect between intelligence and conscientiousness related to baseline levels of German grades (RQ1a). For math grades, results showed a small but statistically (*p* = .046) significant negative interaction term, i.e., a compensatory interaction. Neither intelligence, conscientiousness nor the interaction term predicted the development (i.e., the slope) of grades in both school subjects (RQ1b). 

As for German grades, the control variables of gender and SES predicted the intercept in a statistically significant way, in the sense that girls (as compared to boys) and students from higher-SES families (in comparison to youth from lower-SES families) had better grades. The respective effect sizes were comparable to those of the main predictor variables of fluid intelligence and conscientiousness. No statistically significant effects of the control variables were found in the models with math grades.

## 5. Discussion

Fluid intelligence and conscientiousness have emerged as the most powerful predictors of school grades. Beyond main effects, previous research has yielded first evidence for theoretically plausible interaction effects. A synergistic and a compensatory form of interaction has been proposed. However, most prior studies were cross-sectional, which impeded their explanatory power and many of them relied on older samples solely from the academic track. The aim of the present study was to investigate potential interaction effects of intelligence and conscientiousness in the prediction of school grades in the domains of math and German in a longitudinal sample between age 11 and 15 years comprising students attending different types of school.

### 5.1. Primarily Main Effects of Intelligence and Conscientiousness on School Grades’ Baseline Levels

In our analyses, after controlling for the potential confounding variables of gender and SES, fluid intelligence and conscientiousness predicted baseline levels of school grades. This was more pronounced for math grades than for German grades, which supports our Hypothesis 1a and is in line with previous research (e.g., Brandt et al. 2020b). Ability and personality interacted in the prediction of the initial level of grades in math in a compensatory manner (Research Questions 1a and 2). No interaction effect was found for German grades. 

Students benefitted from being smart and diligent in achieving good grades. With regard to math grades, results suggested the possibility of compensating for low cognitive abilities with a conscientious working style, and vice versa. However, the interaction effect was small and must not be over-interpreted. In terms of German grades, the effects of fluid intelligence and conscientiousness worked in a solely additional manner. The findings contradict the assumption that a conscientious working style amplifies the advantageous effects of high fluid intelligence, with the relation between intelligence and school grades being stronger for the more assiduous students (i.e., synergistic interaction). Our results are partly in line with those of Brandt and Lechner (2022), who analyzed a bigger sample than ours with a comparable age range. Yet, they did not find statistically substantial interaction effects (i.e., after accounting for control variables) on competence levels or competence gains, despite the expectation that effects might be stronger for the prediction of school grades (see also Hübner et al. 2022). Another highly relevant study in this field (Meyer et al. 2022) found small synergistic interaction effects with regard to both German and math grades. Given the very large sample size based on integrative data analysis, Meyer et al.’s study was better positioned to detect even small associations than ours. However, all of their participants were in the academic track.

Other researchers have also found evidence for synergistic intelligence by conscientiousness interaction effects with regard to grades in high school (Bergold and Steinmayr 2018; Sorjonen et al. 2021) and university (Di Domenico and Fournier 2015; Ziegler et al. 2009). Notably, all of these results stem from older and more academic samples than our findings. This pattern could be due to two related reasons. First, the influence of cognitive ability on school grades becomes less relevant and the influence of personality becomes more relevant with age (Lievens et al. 2009). As Bergold and Steinmayr (2018) have noted, this might lead to stronger synergistic interaction effects in older students. In contrast, meta-analytic findings (Poropat 2009) have indicated that the association between academic performance and conscientiousness remains stable over the course of secondary education. Second, it could be that fluid intelligence and conscientiousness amplify each other only in students from the academic track with high levels of both ability and diligence. This might explain why we did not find such effects in our more diverse sample. Restrictions in sample size meant we could not investigate this hypothesis in detail, though it might be an interesting endeavor for future research.

Fluid intelligence and conscientiousness were not related to the intraindividual development of school grades over time in our sample, which contradicts our Hypothesis 1b. The same was true for the interaction term (Research Question 1b). In a longitudinal study covering the same age span as our investigation, Israel et al. (2022) also found a decrease in GPA, but the development of conscientiousness covaried with the changes in school grades. Statistically, the lack of predictive value of conscientiousness (and of fluid intelligence) in our study might stem from the insignificant variances in the slope factors. 

Psychometric quality of self-reported personality is known to differ during the developmental stages of childhood and adolescence (e.g., Soto et al. 2008; Barbaranelli et al. 2008), which might partly explain why we found neither a statistically significant main effect when predicting the development of grades nor an interaction effect with intelligence. In the same vein, personality measured in childhood might not fully overlap with personality in adolescence regarding construct validity and factor structure, thus it might lack predictive power in the longitudinal setting. This aspect should be researched in detail in future studies, however, it is beyond the scope of this study as there are currently no comprehensive measures of personality over several time points available in our data.

In addition to the limited predictive power of conscientiousness on the development of grades in this study, the result pattern was similar for fluid intelligence. We measured fluid intelligence as an indicator of reasoning, which is known to be a good indicator for general cognitive ability (Horn 1988), though less predictive of school performance compared to mixed intelligence measures (combining verbal and non-verbal subtests; e.g., Roth et al. 2015). Indeed, Watkins et al. (2007) found an impact of broader intelligence measures on the development of reading and math competencies. In the same vein, Steinmayr and colleagues (Steinmayr and Spinath 2009; Steinmayr et al. 2019) assessed intelligence via numerical, verbal, and figural reasoning and found intelligence to be predictive of changes in GPA. Besides that, according to Cattell’s investment theory (Cattell 1987) fluid intelligence indicates an initial learning potential to acquire new knowledge, which then is indicated by crystallized intelligence and results in better academic achievement on average, amongst other performance outcomes. However, fluid intelligence itself might not be as strong of a predictor of academic achievement compared to crystallized intelligence or mixed intelligence measures, as it is conceptually more distant from achievement measures than crystallized intelligence. Having said that, fluid intelligence is indicative of underlying potential, however, the development of academic performance might rely more directly on crystallized intelligence as existing learning gaps (i.e., a lack of previously acquired knowledge) are hard to close even if one has high fluid intelligence.

### 5.2. Strengths, Limitations, and Future Directions

Our study was the first to investigate the potential interaction effects of fluid intelligence and conscientiousness on school grades in a longitudinal sample from late childhood to adolescence. Nevertheless, a few limitations need to be acknowledged. First, there was a decrease in sample size between measurement time one and two. This resulted mostly from organizational issues during data collection (see Method section). Although the inclusion of participation at the second measurement time as an auxiliary variable did not change the results, dropout might have reduced the probability of detecting effects. Second, due to the necessities of a large panel study, conscientiousness was measured using a short scale in which not all facets of the conscientiousness dimension were captured explicitly, even though short scales of personality have shown to be comparable to longer assessments (e.g., Rammstedt et al. 2021). Therefore, we were not able to test for differential effects of conscientiousness facets on school grades or observe potential interaction effects with intelligence. Previous research has documented interactions between intelligence and subdomains of conscientiousness, such as Achievement Striving (Ziegler et al. 2009). In the study by Bergold and Steinmayr (2018), all facets except Order interacted with intelligence. In addition, the measure used in this study was not developed for children and young adolescents, however it has shown to be comparable across different age groups from late childhood to adulthood considering psychometrics properties (Brandt et al. 2020a). Notwithstanding these considerations, both fluid intelligence and conscientiousness, but not the interaction term, were associated with the initial levels of school grades with medium effect sizes. This indicates that the measures were conceptually and methodologically suitable to tackle our research questions.

### 5.3. Conclusions

Supporting a long line of research, fluid intelligence and conscientiousness were predictors of school grades in our study on students between late childhood and adolescence. There was a small compensatory interaction between fluid intelligence and conscientiousness in the prediction of math grades. Intelligence and conscientiousness predicted the initial level of school grades, but not the changes therein. Our results underline the general importance of both cognitive abilities and personality for success in school and call into question the generalizability of previously found synergistic interaction effects. These might be more relevant in older and higher-educated samples.

## Figures and Tables

**Table 1:** Sample Characteristics, Descriptive Statistics, McDonald’s Omega (ω), and Sample Sizes (*n*) for all Variables.

		t_1_	t_2_	t_3_
Age	*M*	11.00	13.02	15.07
	*SD*	0.317	0.327	0.345
Conscientiousness	*M*	5.107	-	-
	*SD*	1.097	-	-
	*ω*	.538		
	*n*	1028	-	-
Cognitive abilities	*M*	31.792	-	-
	*SD*	7.603	-	-
	*n*	1025	-	-
Sample size	*N_t_*	1043	749	639

*Note*. Manifest results shown for intelligence and conscientiousness.

**Table 2:** (**a**) Frequencies of grades in math; (**b**) Frequencies of grades in German.

**(a) Math grades**						
	**t_1_**		**t_2_**		**t_3_**	
**Grade**	***n***	**%**	***n***	**%**	***N***	**%**
1	69	11.5	43	9.6	53	12.9
2	246	41.1	170	37.9	139	33.7
3	187	31.2	147	32.7	133	32.3
4	85	14.2	80	17.8	76	18.4
5	12	2.0	9	2	11	2.7
*N*	599		449		412	
**(b) German grades**						
	**t_1_**		**t_2_**		**t_3_**	
**Grade**	***n***	**%**	***n***	**%**	***N***	**%**
1	61	10.2	34	7.5	40	9.7
2	225	37.8	167	36.9	126	30.6
3	236	39.6	187	41.4	167	40.5
4	68	11.4	61	13.5	77	18.7
5	6	1.0	3	0.7	2	0.5
*N*	596		452		412	

*Note*. Lower grades depict higher achievement.

**Table 3:** Manifest correlations among fluid intelligence, conscientiousness, school grades, and the control variables.

Variable	1	2	3	4	5	6	7	8	9
1. Gender	-								
2. SES	−.072								
	(.020)								
3. Gf t_1_	.062	.369							
	(.047)	(<.001)							
4. C t_1_	.073	−.023	.053						
	(.019)	(.454)	(.095)						
5. Math t_1_	−.051	.292	.430	.154					
	(.223)	(<.001)	(<.001)	(<.001)					
6. Math t_2_	.079	.117	.333	.164	.490				
	(.098)	(.014)	(<.001)	(<.001)	(<.001)				
7. Math t_3_	−.003	.110	.297	.110	.469	.588			
	(.946)	(.028)	(<.001)	(.030)	(<.001)	(<.001)			
8. German t_1_	.189	.305	.313	.114	.631	.344	.296		
	(<.001)	(<.001)	(<.001)	(.007)	(<.001)	(<.001)	(<.001)		
9. German t_2_	.201	.116	.203	.132	.299	.565	.378	.332	
	(<.001)	(.015)	(<.001)	(.005)	(<.001)	(<.001)	(<.001)	(<.001)	
10. German t_3_	.279	.077	.161	.212	.342	.467	.497	.414	.609
	(<.001)	(.128)	(.001)	(<.001)	(<.001)	(<.001)	(<.001)	(<.001)	(<.001)

*Note*. *p* values in parentheses. SES = socio-economic status; Gf = fluid intelligence; C = conscientiousness; t_1_–t_3_ = measurement time 1 to 3. Grades were corrected for school type and inverted for interpretability.

**Table 4:** (**a**) Latent growth in grades (math); (**b**) Latent growth in grades (German).

**(a) Math grades**		
**Parameters**	**Est. [95% CI]**	***p***
Latent means		
μ_intercept_	−.004 [−.075; .067]	.914
μ_slope_	−.037 [−.088; .014]	.157
Variances		
σ^2^_intercept_	.422 [.254; .589]	<.001
σ^2^_slope_	.040 [−.054; .134]	.405
Covariances		
σ^2^_intercept, slope_	.014 [−.090; 118]	.793
Model fit		
χ^2^(df)	0.143(1), *p* = .706	
CFI	>.999	
RMSEA [90% CI]	<.001 [.000, .069]	
SRMR	.005	
**(b) German grades**		
**Parameters**	**Est. [95% CI]**	***p***
Latent means		
μ_intercept_	.013 [−.050; .076]	.678
μ_slope_	−.037 [−.084; .009]	.116
Variances		
σ^2^_intercept_	.159 [.030; .289]	.016
σ^2^_slope_	.002 [−.075; .079]	.959
Covariances		
σ^2^_intercept, slope_	.099 [.021; 177]	.016
Model fit		
χ^2^(df)	0.435 (1), *p* = .509	
CFI	>.999	
RMSEA [90% CI]	<.001 [.000, .082]	
SRMR	.008	

*Note*. Unstandardized solution. Grades corrected for school type and inverted for better interpretability. Slope factor loadings were set at 0 (t_1_), 1 (t_2_), and 2 (t_3_). Est. = estimate.

**Table 5:** (**a**) Latent growth curve models predicting baseline level and development of school grades (math); (**b**) Latent growth curve models predicting baseline level and development of school grades (German).

**(a) Math grades**				
	**DV: Intercept**		**DV: Slope**	
	**β**	***p***	**B**	***p***
Model without latent interaction				
C	.235	<.001	−.026	.809
Gf	.593	<.001	−.079	.506
Sex	−.050	.276	.079	.374
SES	.112	.067	−.142	.238
Χ^2^(46) = 76.167, *p* = .003; CFI = .980, RMSEA = .025; SRMR = .031 AIC = 35,269.915; nBIC = 35,344.411				
Latent interaction model				
C	.241	<.001	−.041	.712
Gf	.587	<.001	−.070	.554
C × Gf	−.101	.046	.193	.107
Sex	−.047	.306	.072	.416
SES	.118	.054	−.150	.203
AIC = 35,270.325; nBIC = 35,348.368				
**(b) German grades**				
	**DV: Intercept**		**DV: Slope**	
	**β**	***p***	**B**	***p***
Model without latent interaction				
C	.216	.005	.179	.189
Gf	.472	<.001	−.166	.256
Sex	.292	<.001	.171	.126
SES	.319	<.001	−.195	.168
Χ^2^(46) = 83.215, *p* < .001; CFI = .977, RMSEA = .028; SRMR = .034 AIC = 33,841.671; nBIC = 33,916.168				
Latent interaction model				
C	.218	.004	.178	.177
Gf	.478	<.001	−.171	.236
C × Gf	.096	.237	−.049	.679
Sex	.288	<.001	.170	.122
SES	.312	<.001	−.190	.171
AIC = 35,007.118; nBIC = 35,085.162				

*Note.* Standardized solution. DV = dependent variable; C = conscientiousness; Gf = fluid intelligence; C × Gf = latent interaction between conscientiousness and fluid intelligence; SES = socioeconomic status (composite measure); CFI = comparative fit index; RMSEA = root mean square error of approximation; SRMR = standardized root mean squared residual; AIC = Akaike information criterion; nBIC = Bayesian information criterion corrected for sample size.

## Data Availability

All analyses in this study are based on the TwinLife data release v6.0.0, https://doi.org/10.4232/1.13932. Data have been made available for researchers upon application in the GESIS Data catalogue (DBK) https://search.gesis.org/research_data/ZA6701. Further data documentation including codebooks, methodology reports and questionnaires is available at https://www.twin-life.de/documentation/downloads.

## Supplementary Materials

The following supporting information can be downloaded at: https://www.mdpi.com/article/10.3390/jintelligence11030045/s1, Table S1: Distribution of school types in the current sample; Table S2: Results of Latent Change Models for Grades (Math); Table S3: Results of Latent Change Models for Grades (German); Table S4: Latent change models predicting baseline level and change of grades (math); Table S5: Latent change models predicting baseline level and change of grades (German).

## References

[B1-jintelligence-11-00045] Ackerman Phillip L. (1996). A Theory of Adult Intellectual Development: Process, Personality, Interests, and Knowledge. Intelligence.

[B2-jintelligence-11-00045] Andersen Simon C., Gensowski Miriam, Ludeke Steven G., John Oliver P. (2020). A stable relationship between personality and academic performance from childhood through adolescence. An original study and replication in hundred-thousand-person samples. Journal of Personality.

[B3-jintelligence-11-00045] Bainter Sierra A., Howard Andrea L. (2016). Comparing Within-Person Effects from Multivariate Longitudinal Models. Developmental Psychology.

[B4-jintelligence-11-00045] Barbaranelli Claudio, Fida Roberta, Paciello Marinella, Di Giunta Laura, Caprara Gian Vittorio (2008). Assessing Personality in Early Adolescence Through Self-Report and Other-Ratings a Multitrait-Multimethod Analysis of the BFQ-C. Personality and Individual Differences.

[B5-jintelligence-11-00045] Bardach Lisa, Hübner Nicolas, Nagengast Benjamin, Trautwein Ulrich, von Stumm Sophie (2023). Personality, intelligence, and academic achievement: Charting their developmental interplay. Journal of Personality.

[B6-jintelligence-11-00045] Bergold Sebastian, Steinmayr Ricarda (2018). Personality and Intelligence Interact in the Prediction of Academic Achievement. Journal of Intelligence.

[B7-jintelligence-11-00045] Borghans Lex, Golsteyn Bart H. H., Heckman James J., Humphries John Eric (2016). What Grades and Achievement Tests Measure. Proceedings of the National Academy of Sciences of the United States of America.

[B8-jintelligence-11-00045] Brandt Naemi D., Lechner Clemens M. (2022). Fluid Intelligence and Competence Development in Secondary Schooling: No Evidence for a Moderating Role of Conscientiousness. Journal of Intelligence.

[B9-jintelligence-11-00045] Brandt Naemi D., Becker Michael, Tetzner Julia, Brunner Martin, Kuhl Poldi, Maaz Kai (2020a). Personality Across the Lifespan. European Journal of Psychological Assessment.

[B10-jintelligence-11-00045] Brandt Naemi D., Lechner Clemens M., Tetzner Julia, Rammstedt Beatrice (2020b). Personality, Cognitive Ability, and Academic Performance: Differential Associations Across School Subjects and School Tracks. Journal of Personality.

[B11-jintelligence-11-00045] Brix Jana, Pupeter Monika, Rysina Anna, Steinacker Günter, Schneekloth Ulrich, Baier Tina, Gottschling Juliana, Hahn Elisabeth, Hufer Anke, Kaempfert Merit (2017). A Longitudinal Twin Family Study of the Life Course and Individual Development (TWINLIFE): Data Collection and Instruments of Wave 1 Face-to-Face Interviews (TwinLife Technical Report Series, 05).

[B12-jintelligence-11-00045] Cacioppo John T., Petty Richard E. (1982). The Need for Cognition. Journal of Personality and Social Psychology.

[B13-jintelligence-11-00045] Cattell Raymond B. (1987). Intelligence: Its Structure, Growth and Action.

[B14-jintelligence-11-00045] Chamorro-Premuzic Tomas, Furnham Adrian (2004). A Possible Model for Understanding the Personality—Intelligence Interface. British Journal of Psychology (London, England: 1953).

[B15-jintelligence-11-00045] Cucina Jeffrey M., Peyton Sharron T., Su Chihwei, Byle Kevin A. (2016). Role of Mental Abilities and Mental Tests in Explaining High-School Grades. Intelligence.

[B16-jintelligence-11-00045] Deary Ian J., Strand Steve, Smith Pauline, Fernandes Cres (2007). Intelligence and Educational Achievement. Intelligence.

[B17-jintelligence-11-00045] Deary Ian J., Pattie Alison, Starr John M. (2013). The Stability of Intelligence from Age 11 to Age 90 Years: The Lothian Birth Cohort of 1921. Psychological Science.

[B18-jintelligence-11-00045] Demetriou Andreas, Kazi Smaragda, Spanoudis George, Makris Nikolaos (2019). Predicting School Performance from Cognitive Ability, Self-Representation, and Personality from Primary School to Senior High School. Intelligence.

[B19-jintelligence-11-00045] Denissen Jaap J. A., van Aken Marcel A. G., Penke Lars, Wood Dustin (2013). Self-Regulation Underlies Temperament and Personality: An Integrative Developmental Framework. Child Development Perspectives.

[B20-jintelligence-11-00045] DeYoung Colin G., Sternberg Robert J. (2020). Intelligence and Personality. The Cambridge Handbook of Intelligence.

[B21-jintelligence-11-00045] Di Domenico Stefano I., Fournier Marc A. (2015). Able, Ready, and Willing: Examining the Additive and Interactive Effects of Intelligence, Conscientiousness, and Autonomous Motivation on Undergraduate Academic Performance. Learning and Individual Differences.

[B22-jintelligence-11-00045] Dumfart Barbara, Neubauer Aljoscha C. (2016). Conscientiousness Is the Most Powerful Noncognitive Predictor of School Achievement in Adolescents. Journal of Individual Differences.

[B23-jintelligence-11-00045] Erikson Robert, Goldthorpe John H., Portocarero Lucienne (1979). Intergenerational Class Mobility in Three Western European Societies: England, France and Sweden. The British Journal of Sociology.

[B24-jintelligence-11-00045] Ganzeboom Harry B. G., de Graaf Paul M., Treiman Donald J. (1992). A Standard International Socio-Economic Index of Occupational Status. Social Science Research.

[B25-jintelligence-11-00045] Gerlitz Jean-Yves, Schupp Jürgen (2005). Zur Erhebung der Big-Five-basierten Persönlichkeitsmerkmale im SOEP. Dokumentation der Instrumentenentwicklung BFI-S auf Basis des SOEP-Pretests 2005. https://www.diw.de/documents/publicationen/73/43490/rn4.pdf.

[B26-jintelligence-11-00045] Gottfredson Linda S. (2002). Where and Why G Matters: Not a Mystery. Human Performance.

[B27-jintelligence-11-00045] Gottschling Juliana (2017). Documentation TwinLife Data: Cognitive Abilities. Vol. 02. TwinLife Technical Report Series.

[B28-jintelligence-11-00045] Hagenaars Aldi, de Vos Klaas, Zaidi Asghar, Jenkins Stephen P., Kapteyn Arie, van Praag Bernard M. S. (1998). Patterns of poverty in Europe. The Distribution of Welfare and Household Production: International Perspectives.

[B29-jintelligence-11-00045] Hahn Elisabeth, Gottschling Juliana, Bleidorn Wiebke, Kandler Christian, Spengler Marion, Kornadt Anna E., Schulz Wiebke, Schunck Reinhardt, Baier Tina, Krell Kristina (2016). What Drives the Development of Social Inequality over the Life Course? The German TwinLife Study. Twin Research and Human Genetics: The Official Journal of the International Society for Twin Studies.

[B30-jintelligence-11-00045] Harris-Watson Alexandra M., Kung Mei-Chuan, Tocci Michael C., Boyce Anthony S., Weekley Jeff A., Guenole Nigel, Carter Nathan T. (2022). The Interaction Between Conscientiousness and General Mental Ability: Support for a Compensatory Interaction in Task Performance. Journal of Business and Psychology.

[B31-jintelligence-11-00045] Heaven Patrick C. L., Ciarrochi Joseph (2012). When IQ Is Not Everything: Intelligence, Personality and Academic Performance at School. Personality and Individual Differences.

[B32-jintelligence-11-00045] Hill Patrick L., Jackson Joshua J. (2016). The Invest-and-Accrue Model of Conscientiousness. Review of General Psychology.

[B33-jintelligence-11-00045] Hofer Scott M., Clouston Sean (2014). Commentary: On the Importance of Early Life Cognitive Abilities in Shaping Later Life Outcomes. Research in Human Development.

[B34-jintelligence-11-00045] Horn John L. (1988). Thinking About Human Abilities. Handbook of Multivariate Experimental Psychology.

[B35-jintelligence-11-00045] Horn John L., Cattell Raymond B. (1967). Age Differences in Fluid and Crystallized Intelligence. Acta Psychologica.

[B36-jintelligence-11-00045] Hu Li-tze, Bentler Peter M. (1999). Cutoff Criteria for Fit Indexes in Covariance Structure Analysis: Conventional Criteria Versus New Alternatives. Structural Equation Modeling: A Multidisciplinary Journal.

[B37-jintelligence-11-00045] Hübner Nicolas, Spengler Marion, Nagengast Benjamin, Borghans Lex, Schils Trudie, Trautwein Ulrich (2022). When Academic Achievement (Also) Reflects Personality: Using the Personality-Achievement Saturation Hypothesis (PASH) To Explain Differential Associations Between Achievement Measures and Personality Traits. Journal of Educational Psychology.

[B38-jintelligence-11-00045] Instinske Jana, Rohm Theresa, Mattheus Sophia, Starr Alexandra, Riemann Rainer (2022). Documentation TwinLife Data: Report Cards. v2.0.0. Vol. 04. TwinLife Technical Report Series.

[B39-jintelligence-11-00045] Israel Anne, Brandt Naemi D., Spengler Marion, Göllner Richard, Lüdtke Oliver, Trautwein Ulrich, Wagner Jenny (2022). The Longitudinal Interplay of Personality and School Experiences in Adolescence. European Journal of Personality.

[B40-jintelligence-11-00045] Kingston Paul W., Hubbard Ryan, Lapp Brent, Schroeder Paul, Wilson Julia (2003). Why Education Matters. Sociology of Education.

[B41-jintelligence-11-00045] Klein Andreas, Moosbrugger Helfried (2000). Maximum Likelihood Estimation of Latent Interaction Effects with the LMS Method. Psychometrika.

[B42-jintelligence-11-00045] Laidra Kaia, Pullmann Helle, Allik Jüri (2007). Personality and Intelligence as Predictors of Academic Achievement: A Cross-Sectional Study from Elementary to Secondary School. Personality and Individual Differences.

[B43-jintelligence-11-00045] Lang Volker, Kottwitz Anita (2020). The Socio-Demographic Structure of the First Wave of the TwinLife Panel Study: A Comparison with the Microcensus. Methods, Data, Analyses.

[B44-jintelligence-11-00045] Lesaar Sabrina, Prussog-Wagner Angela, Hess Doris (2020). TwinLife Survey Methodology and Fieldwork Outcomes. Face-to-Face Survey of Wave 2 (F2F 2a/b). v1.0.0. Vol. 10. TwinLife Technical Report Series.

[B45-jintelligence-11-00045] Lievens Filip, Ones Deniz S., Dilchert Stephan (2009). Personality Scale Validities Increase Throughout Medical School. The Journal of Applied Psychology.

[B46-jintelligence-11-00045] Litman Jordan A. (2008). Interest and Deprivation Factors of Epistemic Curiosity. Personality and Individual Differences.

[B47-jintelligence-11-00045] Lüttinger Paul, König Wolfgang (1988). Die Entwicklung Einer International Vergleichbaren Klassifikation Für Bildungssysteme [Development of a Internationally Comparable Classification for Educational Systems]. ZUMA Nachrichten.

[B48-jintelligence-11-00045] Maier Norman R. F. (1965). Psychology in Industry.

[B49-jintelligence-11-00045] Mammadov Sakhavat (2022). Big Five Personality Traits and Academic Performance: A Meta-Analysis. Journal of Personality.

[B50-jintelligence-11-00045] Maslowsky Julie, Jager Justin, Hemken Douglas (2015). Estimating and Interpreting Latent Variable Interactions: A Tutorial for Applying the Latent Moderated Structural Equations Method. International Journal of Behavioral Development.

[B51-jintelligence-11-00045] McCrae Robert R., Costa Paul T. (1987). Validation of the Five-Factor Model of Personality Across Instruments and Observers. Journal of Personality and Social Psychology.

[B52-jintelligence-11-00045] Meyer Jennifer, Lüdtke Oliver, Schmidt Fabian T. C., Fleckenstein Johanna, Trautwein Ulrich, Köller Olaf (2022). Conscientiousness and Cognitive Ability as Predictors of Academic Achievement: Evidence of Synergistic Effects from Integrative Data Analysis. European Journal of Personality.

[B53-jintelligence-11-00045] Moutafi Joanna, Furnham Adrian, Paltiel Laurence (2004). Why Is Conscientiousness Negatively Correlated with Intelligence?. Personality and Individual Differences.

[B54-jintelligence-11-00045] Murray Aja L., Johnson Wendy, McGue Matt, Iacono William G. (2014). How Are Conscientiousness and Cognitive Ability Related to One Another? A Re-Examination of the Intelligence Compensation Hypothesis. Personality and Individual Differences.

[B55-jintelligence-11-00045] Muthén Linda K., Muthén Bengt O. (1998–2017). Mplus User’s Guide.

[B56-jintelligence-11-00045] Neisser Ulric, Boodoo Gwyneth, Bouchard Thomas J., Boykin A. Wade, Brody Nathan, Ceci Stephen J., Halpern Diane F., Loehlin John C., Perloff Robert, Sternberg Robert J. (1996). Intelligence: Knowns and Unknowns. American Psychologist.

[B57-jintelligence-11-00045] Nießen Désirée, Danner Daniel, Spengler Marion, Lechner Clemens M. (2020). Big Five Personality Traits Predict Successful Transitions from School to Vocational Education and Training: A Large-Scale Study. Frontiers in Psychology.

[B58-jintelligence-11-00045] Peng Peng, Wang Tengfei, Wang Cuicui, Lin Xin (2019). A Meta-Analysis on the Relation Between Fluid Intelligence and Reading/mathematics: Effects of Tasks, Age, and Social Economics Status. Psychological Bulletin.

[B59-jintelligence-11-00045] Poropat Arthur E. (2009). A Meta-Analysis of the Five-Factor Model of Personality and Academic Performance. Psychological Bulletin.

[B60-jintelligence-11-00045] Rammstedt Beatrice, Lechner Clemens M., Danner Daniel (2021). Short Forms Do Not Fall Short: A Comparison of Three (Extra-)Short Forms of the Big Five. European Journal of Psychological Assessment.

[B61-jintelligence-11-00045] Rindermann Heiner (2006). Was messen internationale Schulleistungsstudien?. Psychologische Rundschau.

[B62-jintelligence-11-00045] Roberts Brent W., DelVecchio Wendy F. (2000). The Rank-Order Consistency of Personality Traits from Childhood to Old Age: A Quantitative Review of Longitudinal Studies. Psychological Bulletin.

[B63-jintelligence-11-00045] Roberts Brent W., Walton Kate E., Viechtbauer Wolfgang (2006). Patterns of Mean-Level Change in Personality Traits Across the Life Course: A Meta-Analysis of Longitudinal Studies. Psychological Bulletin.

[B64-jintelligence-11-00045] Roth Bettina, Becker Nicolas, Romeyke Sara, Schäfer Sarah, Domnick Florian, Spinath Frank M. (2015). Intelligence and School Grades: A Meta-Analysis. Intelligence.

[B65-jintelligence-11-00045] Sackett Paul R., Gruys Melissa L., Ellingson Jill E. (1998). Ability-Personality Interactions When Predicting Job Performance. Journal of Applied Psychology.

[B66-jintelligence-11-00045] Shaw Philip, Greenstein Deanna, Lerch Jason, Clasen Liv, Lenroot Roshel, Gogtay Nitin, Evans Alan C., Rapoport Judith, Giedd Jay (2006). Intellectual Ability and Cortical Development in Children and Adolescents. Nature.

[B67-jintelligence-11-00045] Sorjonen Kimmo, Wallin Alma Sörberg, Falkstedt Daniel, Melin Bo (2021). Personality Trait by Intelligence Interaction Effects on Grades Tend to Be Synergistic. BMC Psychology.

[B68-jintelligence-11-00045] Soto Christopher J., Tackett Jennifer L. (2015). Personality Traits in Childhood and Adolescence. Current Directions in Psychological Science.

[B69-jintelligence-11-00045] Soto Christopher J., John Oliver P., Gosling Samuel D., Potter Jeff (2008). The Developmental Psychometrics of Big Five Self-Reports: Acquiescence, Factor Structure, Coherence, and Differentiation from Ages 10 to 20. Journal of Personality and Social Psychology.

[B70-jintelligence-11-00045] Steinmayr Ricarda, Spinath Birgit (2009). The Importance of Motivation as a Predictor of School Achievement. Learning and Individual Differences.

[B71-jintelligence-11-00045] Steinmayr Ricarda, Weidinger Anne F., Schwinger Malte, Spinath Birgit (2019). The Importance of Students’ Motivation for Their Academic Achievement—Replicating and Extending Previous Findings. Frontiers in Psychology.

[B72-jintelligence-11-00045] Tetzner Julia, Becker Michael, Brandt Naemi D. (2020). Personality-Achievement Associations in Adolescence-Examining Associations Across Grade Levels and Learning Environments. Journal of Personality.

[B73-jintelligence-11-00045] van Iddekinge Chad H., Aguinis Herman, Mackey Jeremy D., DeOrtentiis Philip S. (2018). A Meta-Analysis of the Interactive, Additive, and Relative Effects of Cognitive Ability and Motivation on Performance. Journal of Management.

[B74-jintelligence-11-00045] von Stumm Sophie, Plomin Robert (2015). Socioeconomic Status and the Growth of Intelligence from Infancy Through Adolescence. Intelligence.

[B75-jintelligence-11-00045] von Stumm Sophie, Chamorro-Premuzic Tomas, Ackerman Phillip L., Chamorro-Premuzic Tomas (2011). Re-Visiting Intelligence–personality Associations: Vindicating Intellectual Investment. The Wiley-Blackwell Handbook of Individual Differences.

[B76-jintelligence-11-00045] Watkins Marley W., Lei Pui-Wa, Canivez Gary L. (2007). Psychometric Intelligence and Achievement: A Cross-Lagged Panel Analysis. Intelligence.

[B77-jintelligence-11-00045] Weiß Rudolf H. (2006). CFT 20-R: Grundintelligenztest Skala 2—Revision.

[B78-jintelligence-11-00045] Wrzus Cornelia, Roberts Brent W. (2017). Processes of Personality Development in Adulthood: The TESSERA Framework. Personality and Social Psychology Review: An Official Journal of the Society for Personality and Social Psychology.

[B79-jintelligence-11-00045] Yu Huihui, McCoach D. Betsy, Gottfried Allen W., Gottfried Adele Eskeles (2018). Stability of Intelligence from Infancy Through Adolescence: An Autoregressive Latent Variable Model. Intelligence.

[B80-jintelligence-11-00045] Zhang Jing, Ziegler Matthias (2015). Interaction Effects Between Openness and Fluid Intelligence Predicting Scholastic Performance. Journal of Intelligence.

[B81-jintelligence-11-00045] Ziegler Matthias, Knogler Maximilian, Bühner Markus (2009). Conscientiousness, Achievement Striving, and Intelligence as Performance Predictors in a Sample of German Psychology Students: Always a Linear Relationship?. Learning and Individual Differences.

