# Supplementary material for: Is There Evidence for Intelligence-by-Conscientiousness Interaction in the Prediction of Change in School Grades from Age 11 to 15 Years?
Source: J Intell. 2023 Feb 25;11(3):45. doi: 10.3390/jintelligence11030045 (PMC10058778; doi:10.3390/jintelligence11030045)
Supplement: Supplementary file 1 [file jintelligence-11-00045-s001.zip › jintelligence-2142274-supplementary.pdf]

Journal of
Intelligence

Table S1

Distribution of school types in the current sample

School type	Measurement time		
	t_1	t_2	t_3
Elementary school	46.7	3.3	0.2
Lower secondary school	0.5	0.8	1.2
Intermediate secondary school	5.8	11.3	11.8
Integrated lower and intermediate school	3.6	5.0	6.1
Comprehensive school	10.7	24.0	20.6
Waldorf school	1.4	1.5	1.7
Upper secondary school	27.5	51.7	54.8
School for special needs	1.4	1.7	2.4
Other type of school	2.4	0.8	1.2

Note. Percentages are depicted.

Table S2

Results of Latent Change Models for Grades (Math)

Parameters	Est.	[95% CI]	p
Latent means			
$\mu_{Baseline}$	-.004	[-.075; .067]	.911
μ_{Change}	-.037	[-.088; .014]	.154
Variances			
$\sigma^2_{Baseline}$	.478	[.366; .590]	<.001
σ^2_{Change}	.057	[-.001; .114]	.054
Covariances			
$\sigma^2_{Baseline, Change}$	-.019	[-.088; .014]	.563
Model fit			
χ^2(df)		1.148 (3), p = .765	
CFI		>.999	
RMSEA [90% CI]		<.001 [.000, .041]	
SRMR		.005	

Note. Unstandardized solution from the constant change model. Grades corrected for school type and inverted for better interpretability. Est. = estimate.

Table S3

Results of Latent Change Models for Grades (German)

Parameters	Est.	[95% CI]	*p*
Latent means			
$\mu_{Baseline}$	.005	[-.075; .067]	.869
μ_{Change}	-.031	[-.088; .014]	.198
Variances			
$\sigma^2_{Baseline}$	.365	[.366; .590]	<.001
σ^2_{Change}	.052	[-.001; .114]	.040
Covariances			
$\sigma^2_{Baseline, Change}$	-.018	[-.088; .014]	.514
Model fit			
χ^2(df)		20.658 (3), p = .765	
CFI		<.001	
RMSEA [90% CI]		<.001 [.000, .041]	
SRMR		.060	

Note. Unstandardized solution from the constant change model. Grades corrected for school type and inverted for better interpretability. Est. = estimate.

Table S4

Latent change models predicting baseline level and change of grades (math)

	DV: Baseline		DV: Growth	
	β	*p*	β	*p*
	Conditional models without latent interaction			
C	.241	<.001	-.031	.810
Gf	.606	<.001	-.095	.501
(Sex)	-.050	.290	.094	.366
(SES)	.114	.070	-.171	.214
	$X^2(48)$ 76.868, *p* =.006; CFI = .981, RMSEA = .024; SRMR = .031 AIC = 35266.456; nBIC = 35464.451			
	Latent interaction model			
C	.246	<.001	-.047	.717
Gf	.600	<.001	-.085	.547
C x Gf	-.104	.043	.227	.098
(Sex)	-.047	.321	.086	.409
(SES)	.119	.056	-.181	.179
	AIC = 35266.840; nBIC = 35474.734			

Note. Standardized solution from the constant change model. DV = dependent variable; C= conscientiousness; Gf = fluid intelligence;
C x Gf = latent interaction between conscientiousness and fluid intelligence; SES = socioeconomic status (composite measure);
CFI = comparative fit index; RMSEA = root mean square error of approximation;
SRMR = standardized root mean squared residual; AIC = Akaike information criterion; nBIC = Bayesian information criterion corrected for sample size. Grades were corrected for school type and inverted for interpretatbility.

Table S5
Latent change models predicting baseline level and change of grades (German)

	DV: Baseline		DV: Change	
	β	*p*	β	*p*
**Baseline model**				
C	.188	.002	.158	.258
Gf	-391	<.001	-.200	.186
(Sex)	.254	<.001	.133	.231
(SES)	.282	<.001	-.270	.060
X^2(48 = 92.473, *p* <.001; CFI = .969, RMSEA = .030; SRMR = .038 AIC = 35013.914; nBIC = 35211.908				
**Latent interaction model**				
C	.191	.002	.160	.241
Gf	.400	<.001	-. 209	.169
C x Gf	.088	.181	-.082	.539
(Sex)	.252	<.001	.135	.222
(SES)	.276	<.001	-. 265	. 064
AIC = 35016.273; nBIC = 35224.167				

Note. Standardized solution from the constant change model. DV = dependent variable; C= conscientiousness; Gf = fluid intelligence;
C x Gf = latent interaction between conscientiousness and fluid intelligence; SES = socioeconomic status (composite measure);
CFI = comparative fit index; RMSEA = root mean square error of approximation;
SRMR = standardized root mean squared residual; AIC = Akaike information criterion; nBIC = Bayesian information criterion corrected for sample size. Grades were corrected for school type and inverted for interpretability.
